# Carbon nanotube-reduced graphene oxide fiber with high torsional strength from rheological hierarchy control

**DOI:** 10.1038/s41467-020-20518-0

**Published:** 2021-01-15

**Authors:** Wonsik Eom, Eunsong Lee, Sang Hoon Lee, Tae Hyun Sung, Adam J. Clancy, Won Jun Lee, Tae Hee Han

**Affiliations:** 1 Department of Organic and Nano Engineering, Hanyang University, Seoul, 04763 Republic of Korea; 2 Department of Electrical Engineering, Hanyang University, Seoul, 04763 Republic of Korea; 3 Department of Chemistry, University College London, London, WC1H 0AJ UK; 4 Department of Fiber System Engineering, Dankook University, Yongin-si, 16890 Republic of Korea; 5 Human-Tech Convergence Program, Hanyang University, Seoul, 04763 Republic of Korea

**Keywords:** Condensed-matter physics, Nanoscale materials, Soft materials

## Abstract

High torsional strength fibers are of practical interest for applications such as artificial muscles, electric generators, and actuators. Herein, we maximize torsional strength by understanding, measuring, and overcoming rheological thresholds of nanocarbon (nanotube/graphene oxide) dopes. The formed fibers show enhanced structure across multiple length scales, modified hierarchy, and improved mechanical properties. In particular, the torsional properties were examined, with high shear strength (914 MPa) attributed to nanotubes but magnified by their structure, intercalating graphene sheets. This design approach has the potential to realize the hierarchical dimensional hybrids, and may also be useful to build the effective network structure of heterogeneous materials.

## Abstract (web summary)

Fibers with high torsional strength are of practical interest for artificial muscles, electric generators, actuators, etc. Here, the authors optimize torsional strength by overcoming rheological thresholds of nanocarbon (nanotube/graphene oxide) dopes.

## Introduction

The assembly of nanomaterials into well-ordered, hierarchical structures is established as a route to improve properties^1–3^. The low-dimension allotropes of carbon, carbon nanotubes (CNTs) and graphene, are common nanomaterials for structural applications due to their high intrinsic mechanical properties, low densities, and supplementary functional properties such as high thermal/electrical conductivities. In the absence of hierarchical organization, their properties are significantly inferior to the intrinsic materials, partly due to the Griffith criterion, but also the presence of mechanically inferior agglomerates, poor alignment versus the shear direction, and presence of voids.

One particularly promising approach to create ordered nanomaterial fibers is to exploit well-established microscale fluid control using solutions/dispersions of nanomaterials to control the assembled architecture to manipulate the nanoscale functionality^4–8^. In particular, liquid-phase nanomaterials may be assembled into fibers through extrusion of a nanomaterial dope with concurrent removal of the solvent to provide a solid ‘filament’ of pure nanomaterial (or composite fiber if mixed with an additional continuous solid phase, typically polymer). The nanocarbons family of CNTs, graphene oxide (GO), and reduced graphene oxide (rGO) are classic species used for fibers^3^, owing to their superlative intrinsic mechanical properties. The use of hybridization of mixed species has also been demonstrated to provide mutually beneficial reinforcement and/or deformation mechanics, although this typically occurs for composite fibers not pure nanocarbon fibers^9^. The use of mixed large and small graphene flakes has been shown to create improved pure-graphene fibers^10^, although performance is limited versus the state-of-the-art pure-CNT fibers^11^. Control over fiber microstructure may be gained by manipulating the forces applied to the solution during extrusion (e.g. shear-alignment/sheath solvents)^12^ and during solvent removal (e.g. drying in electrospinning, or solution dynamics during coagulation)^13^.

The dope itself has significant impact on the final properties too, with solvent^14^, nanomaterial surface chemistry^15^, concentration^16^, degree of dispersion^17^, and additives^18^ all providing variables which may be used to control the final properties. However, while microscale solution control provides a degree of structure, nanoscale heterogeneity generally limits the long-range orientational order, generating grain boundaries (bulges/slides) or pores during shear-induced flow. These defects result in fluctuation of the viscoelastic properties during the transformation from the liquid to solid state^18–20^, causing structural distortion in the form of coupling density fluctuations and deformation fields. Understanding the intrinsic properties of the dope constituents reorienting during application of shear^21,22^ should allow for better understanding of how to control the fiber microstructure during this transition.

While most contemporary nanomaterial fibers concentrate on the (easily measured) tensile properties, for many applications, the torsional strength is of critical importance. The torsional strength (~shear strength) is the ability of a material to withstand a twisting load (~torque). Fibers consisting of pure CNTs have shown particular promise in torsional applications, with much interest in utilization for functional applications, such as sensors and actuator^23–25^, but is poorly understood in the case of nanomaterial-based materials due to the complicated nature of the applied moments in torsion. In principle, unlike axial loads which produce a uniform (or average) stress on the cross-section of a material, torque produces a stress distribution across the cross-section (caused by a shear strain difference along the radius). In multicomponent systems, effective stress distribution requires the practical arrangement of constituents often described in terms of matrix and linkers. The linker can sustain torsional moments by distributing the load evenly around the inner perimeter of the fiber. From this perspective, lyotropic liquid crystals^26^, which are mesogenic phases of matter that combine liquid fluidity with crystalline solid properties, provide an interesting model platform as a matrix to manipulate the kinetics of solidification on a finite timescale. The transition from isotropic liquid to anisotropic fiber during spinning is associated with dynamic flow of the microscopic structure, providing a handle to manipulate the final structure.

Here, rGO fibers are created, with and without CNT hybridization to provide pure nanocarbon fibers. Through the precise addition of rigid nanotubes^27,28^, the dope properties are modified and by understanding the dope properties (treated as a glassy flow) and applying suitable shear force, constituent alignment is controlled. The created fibers have dramatically improved microstructures, with high torsional performance arising from their structure.

## Results

### Wet spinning applying control of flow dynamics and elastic asymmetry

**Fig. 1:**
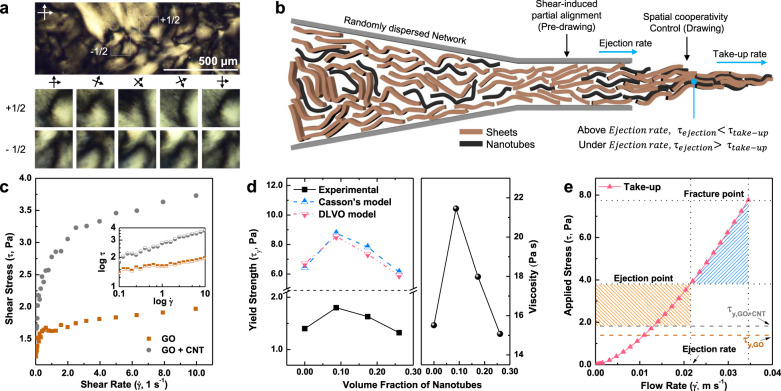
Wet spinning applying control of flow dynamics and elastic asymmetry. **a** Schlieren texture and disclination of GO and CNTs revealed through polarized optical microscopy. **b** Schematic illustration of a wet spinning. **c** Yield strengths of the GO dispersion and mixture solution of GO and CNTs obtained in the steady mode at 0.1 s^−1^. **d** Experimental yield strengths in the steady mode and the yield strengths calculated from Casson and DLVO models of sheets with nanotube dispersion in the oscillatory mode. **e** Applied stress induced by different ejection and take-up rates. The blue region represents the range in which an alignment by drawing process is realized.

Nanocarbon fibers are synthesized here through coagulation spinning of a fiber from a dope to form a gel, partially aligned through the pre-drawing effect during injection through the die (Fig. 1). After the injection of the nanocarbon dope, the solidifying components are subjected to dynamic flow which promotes rearrangement/reorientation. However, the motion under flow of a given nanomaterial is impacted by co-solvated species in a manner reminiscent of spatial cooperativity^21^ seen in glassy flows (Fig. 1a, b and Supplementary Fig. 2) which impacts the viscoelastic properties of the flowing materials. This behavior may be best illustrated by the dynamic rheological changes seen upon altering the geometry of a fraction of the nanocarbons, by replacing GO with CNTs. The similarity in behavior to glassy flows is surprising given the low concentration of GO (5 mg mL^−1^), but can be rationalized through considering the GO’s large hydrodynamic radius (Supplementary Fig. 3). As reorienting behavior and viscoelastic dope response are intrinsically linked, alignment can be maximized (and hierarchical structure controlled) by competitive control of the fluid dynamics of the dope under flow, and the elastic recovery in the (glassy-like) flow of these colloidal nanomaterials. The dynamic yield strength (i.e. critical stress to initiate the flow) of the dope provides direct insight into the interspecies cooperation of these nanocarbon building blocks^21^. Specifically, this information was sought to address three questions: (i) how to drive a smooth liquid–solid transition from dope to fiber under sufficient stress with non-linear flow characteristics, (ii) how to rearrange the constituents in shear flows, and (iii) how to predict the necessary external force to alter the velocity profiles in shear flows to align the constituents.

Upon addition of CNTs to the GO dope, the apparent rheological yield strength initially increased (Fig. 1c, d), from 1.39 Ga for the pure GO dope to 1.82 Pa for a CNT volume fraction (*V*_f_) of 0.087, as is expected upon introduction of an elastic component. The colloid yield strength (measured from a flow curve diagram, Fig. 1c inset) indicated that the addition of CNTs led to a higher shear stress threshold for the rearrangement of the species, even at a low shear rate (0.1 s^−1^). This behavior can be attributed to CNTs linking GO sheets to build a stronger solute-network structure than a homogeneous, pure-GO system^22^. However, at higher CNT volume fractions (Fig. 1d), the shear stress decreases toward the GO solution properties, attributed to increasing degree of aggregation of the CNTs (as seen in x-ray scattering, cryofractures, and x-ray tomography in the final fibers, vide infra). Agglomeration replaces the strong GO–CNT interactions with weaker CNT–CNT interactions, and begins to disrupt the GO dispersion, as seen by the loss of liquid crystallinity at *V*_f_ > 0.26. The initial increase in rheological shear strength trend was modeled by two different structural rheological models: (i) Casson^29,30^ and (ii) DLVO^31^ (see SI for further details). Briefly, the Casson and DLVO models predict that the introduction of CNTs may increase the yield strength of GO solutions by factors of approximately 1.36× and 1.27×, respectively (Supplementary Tables 1–3), matching the 1.31× increase seen here. The difference in absolute strength is attributed to experimental values being obtained through motion in flow direction, while theoretical values are calculated based on the oscillatory motion, which incorporates sinusoidal shear deformation.

Importantly, the underlying mechanism of measurements sheds light on the behavior of the materials during processing. The yield strength obtained from (unidirectional) flow indicates the minimum force required to initiate reorganization during the fluid flow through a spinneret, treated as a single system (i.e. treating components concomitantly). In contrast, the yield strength obtained through the oscillatory motion allows estimation of the level of bonding of constituents based on repulsive and attractive forces, reflective of the stresses for separate components in a heterogeneous fluid. When the fluid ejected from the nozzle, it is taken up by rollers at a defined rate faster than the extrusion velocity, subjecting the fluid to a tensile stress. The applied stress necessary for fracture (7.82 Pa) is consistent with the Casson (8.77 Pa) and DLVO (8.54 Pa) models, due to the segregation of constituents during the transition from fluid to solid (Fig. 1e). To provide the necessary stress to facilitate the reorientation without breaking the dope (i.e. 1.82 Pa < stress < 7.82 Pa), the applied stress was set to 4.37 Pa with an additional take-up force (using a take-up rate of 0.021 m s^−1^), which is higher than the original ejection force (3.70 Pa, see SI for details). The introduction of rheologically calculated drawing force impacted the microstructures not only through alignment, but also inter-layer GO distance, and specific density after the transition from a colloidal dispersion to a solid to create highly aligned and CNT-reinforced graphene fibers. Both drawn pure GO (D-GF) and CNT/GO hybrid (D-HF, *V*_f_ = 0.174) fibers were spun, alongside controls lacking the controlled take-up (GF and HF, respectively).

### Highly oriented and compactly integrated structure of D-HF

**Fig. 2:**
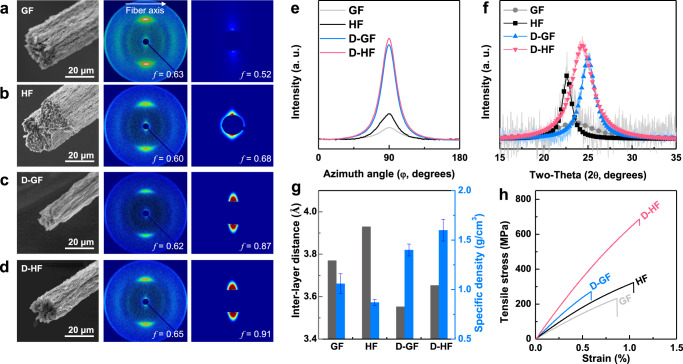
Highly oriented and compactly integrated structures of fibers. Morphology, WAXS, and SAXS patterns of **a** rGO-only fibers (GF), **b** hybrid fibers (HF) prepared without a take-up process (drawing), **c** rGO-only fibers (D-GF), and **d** hybrid fibers (D-HF) with drawing. **e** Azimuthal plot representing the Herman’s orientation factor from SAXS. **f** XRD pattern showing the distribution of the inter-layer distances. **g** Inter-layer distance and specific density. **h** Tensile stress–strain curve of the fibers.

All samples were chemically reduced in a diluted hydroiodic acid (HI) solution to transform the GO into rGO, without harsh reduction routes which would lead to loss of framework carbons in the form of evolved CO/CO_2_, and potential disruption of the fiber structure from internally evolved gas^32^. The cross-sectional areas of D-GF (245.9 ± 30.6 μm^2^) and D-HF (235.8 ± 28.3 μm^2^), as measured by SEM of cryofractures (Fig. 2a–d) were remarkably smaller than those of GF (397.2 ± 4.8 μm^2^) and HF (727.9 ± 99.6 μm^2^). It is noted that all measured cross-sections showed rough, asymmetric circumferences. It could be seen that CNTs were evenly distributed in the cross-section of the D-HF (Supplementary Fig. 4a). The alignment of the components at short and long length scales was quantified as the corresponding Herman’s orientation factor (*f*, see SI) using a characteristic full-width-half-maximum in wide and small angle x-ray scattering (WAXS/SAXS), respectively (Fig. 2a–d).

In the absence of controlled take-up, i.e. applying insufficient force to the dope to introduce drawing effect, the presence of CNTs led to lower alignment (*f*_HF_ = 0.60 WAXS) than the pure GO control (*f*_GF_ = 0.63) in Fig. 2e. This effect follows the expected trend of an additive (CNTs) in a liquid crystal disrupting intrinsic alignment, limiting final fiber alignment. The reduction in alignment is more dramatically seen for the (long range ordering) SAXS, with the short-range local ordering presumably dominated by the liquid crystalline rGO domains. The use of controlled take-up reverses this trend, however, with a higher orientation for D-HF (*f*_D-HF_ = 0.65 and 0.91), than D-GF (*f*_D-GF_ = 0.62 and 0.87). This increase in alignment indicates that the presence of CNTs aided the alignment of the GO sheets, confirming the CNTs aided the reorientation by increasing GO mobility during take-up to provide the aligned network structure.

In addition to alignment, the rGO inter-layer distancing was impacted by processing and presence of CNTs, as measured by x-ray diffraction (XRD, Fig. 2f). Without rheological control, the GF showed separation of 3.77 Å (2*θ* = 23.56°, Cu Kα) with a wide distribution, while addition of CNTs for HF widened to 3.93 Å (22.58°) but led to a significantly narrower peak. The CNTs can be seen to interact with rGO sheets in a heterogeneous fashion, even at the cost of alignment (as seen in WAXS/SAXS). The use of rheological control led to a decrease in spacings, as seen in previous graphene fibers^33^. Here both pure D-GF (3.55 Å, 25.03°) and hybrid D-HF (3.65 Å, 24.34°) demonstrated decreased inter-layer distances, contributing to higher packing densities and corresponding increases in the specific density and lower porosities (Fig. 2g and Supplementary Fig. 4b). Due to the small total weight of fiber produced, classic BET measurements were not possible to probe pore quantity and structure; however, the reduced porosity in D-HF and D-GF compared to HF and GO was confirmed using x-ray nano-cryotomography data (Supplementary Fig. 5)^34^ and cryotomographic TEM micrographs of the fibers (Supplementary Fig. 6). Interestingly, the presence of CNTs led to significantly higher tensile strength in D-HF (690 ± 76 MPa) compared to that of D-GF (272 ± 74 MPa), shown in Fig. 2h. The origin of this increase in tensile properties may be attributed to the greater alignment of nanocarbons in the fibers, and their tolerant network structure during stretching deformation^35^. As seen for previous graphene-based fibers^33^, controlled take-up led to an increase compared to the initial fiber (GF = 229 ± 40 MPa), and this effect was also present for hybrid systems (HF 325 ± 92 MPa). The behavior of all fibers was similar under tension, with all materials failing in a brittle manner, similar to other pure-nanocarbon fibers^3^.

### Torsional performance of D-HF

**Fig. 3:**
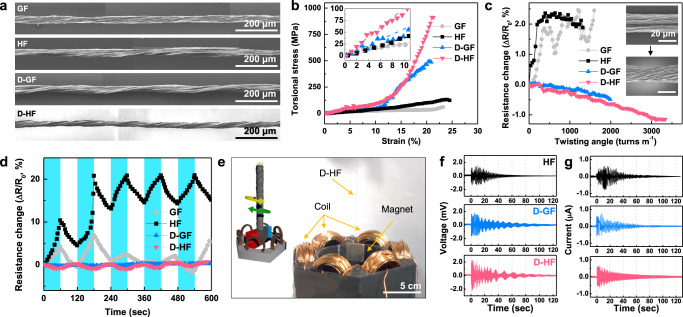
Torsional performance of fibers. **a** Twisted morphology of GF, HF, D-GF, and D-HF. The fibers were twisted by two-thirds the number of breakages (GF: 1800, HF: 1300, D-GF: 1800, D-HF: 2500 turns m^−1^). **b** Torsional stress–strain curves of the fibers. The inset shows a close-up of the strain region from 0 to 10%. **c** Resistance change as a result of torsional deformation until the breakage points. The inset shows a shrunken D-GF fiber by twisting. **d** Variation of resistance during cycle testing of repeating twisting (blue region) and untwisting (white region). **e** Scheme of the designed torsional current energy estimating device based on D-HF. The device contains six copper coils around the fiber, along with a magnet. When the twisted fiber is released, the magnet can reversibly be rotated within the surrounding copper coils to generate electricity. **f** Open-circuit voltage and **g** short-circuit current of the device with HF, D-GF, and D-HF.

Torsional strength refers to the ability of a material to withstand a twisting load and is closely related to the shear strength, which was expected to increase with better controlled microstructure. Here, a bespoke setup was created to measure the torsional properties with real-time optical monitoring through modification of a commercial rheometer (Supplementary Figs. 7, 8 and Supplementary Movie 1). D-HF greatly resisted the torque applied during twisting (914 ± 61 MPa, Fig. 3a, b), indicating effective transmission of stress between components, owing to the well-integrated CNTs in the rGO network, high degree of alignment, and increased density facilitating greater inter-component contact. The role of the CNTs can be seen from the significant increase versus D-GF (496 ± 42 MPa) which itself was almost a tenfold increase over GF (53 ± 8 MPa). Even in the absence of rheologically controlled processing, the presence of CNTs allowed a significant increase in torsional strength (HF, 132 ± 53 MPa).

To ascertain these effects, stress–torsional strain curves of all samples were investigated in detail (Fig. 3b and Supplementary Table 4). When the number of twists (i.e., torsional strain) increased for GF and HF, the stress was linearly proportional to strain until failure, indicating applied stress is stored elastically, as supported by the absence of volume change upon application of torsion (Supplementary Fig. 7c). In principle, torsional stress leads to a volume reduction from concomitant rearrangement of constituents; however, GF and HF lack mobility of constituents in their microstructures, so showed no such rearrangements prior to fracture. In contrast, D-GF and D-HF deviated from a linear stress–strain behavior and established an exponential stress/strain dependence. This non-linearity indicates stress-induced deformation from constituent rearrangement of its internal molecular or microscopic structure^36^.

Upon closer inspection, the observed exponential dependence of stress on strain can be interpreted considering similar synergistic effects to those in the dope which dictated structure during spinning. The extension of the concept can be envisioned as the flow-induced alignment during fiber spinning being frozen upon solidification, but resumed under the ‘flow’ of torsional stress. Similar torsional-strain stiffening has been pronounced for many natural systems including proteins, collagen, cytoskeletal networks of actin, and intermediate filaments^37^. Notably, such bio-systems consist of a heterogeneous system exhibiting a united contraction mode of short, stiff ‘chains’ and a softer matrix, with high intermolecular adhesion^38^. In our case for D-HF, the strong network is between softer rGO matrix and short stiff ‘chains’ of CNTs.

The electrical resistance of the fibers changed under torsion, with behavior trend dependent on whether the fiber was ‘aligned’ through rheologically controlled take-up. The in situ measured resistance (Fig. 3c) of poorly aligned GF and HF increase with increasing number of twists (i.e. increasing torsion), attributed to the production of fractures and cracks at the boundaries between constituents. The breaking of a brittle microstructure under torsional strain increasing electrical resistance is often observed in conventional material systems^39^. Cycling of the torsion (i.e. twisting/untwisting at 10 r.p.m., Fig. 3d) also shows a high degree of hysteresis, reflecting the high degree of structural distortion and low inter-nanocarbon adhesion strength^40^. The irreversible and irregular resistance changes seen for HF and GF indicate the unrecoverable fractures have occurred as can be seen at the surface (Supplementary Figs. 9 and 10). After initial cracking from the first two cycles, twisting (re)opened these cracks, which close during reversal of the twisting. The resistance never recovers to its pre-cracked level; however, no new cracks are formed in subsequent cycles^41^. In stark contrast, in the aligned D-GF and D-HF, the ability to rearrange constituents under torsion allows the fiber to condense with twisting, increasing contact between the intrinsically conducting species, shortening the conductive pathway and mildly decreasing resistance (Fig. 3c and Supplementary Figs. 11 and 12)^41,42^.

To uncouple the contribution to the elastic potential energy from CNTs, the potential energy in the fibers was transformed into electrical energy with a customized motor^43,44^. This approach allowed monitoring of both electrical pathway formation/decay at the microscale, and the time-dependent resistivity changes upon stress relaxation. Simply, a magnetic paddle was attached to the end of the twisted fiber, which could rotate within a custom dynamo (Fig. 3e). The open-circuit voltage (*V*_OC_) and short-circuit current (*I*_SC_) in *I*–*V* curves quantify stored power and residual stress release with the torsional action, respectively (Fig. 3f, g). In principle, V_OC_ is proportional to the rotational speed and provides an absolute measure of the elastic potential power stored in the microstructure. The rotational speed was proportional to torsional modulus in a range of elastic elongation (Fig. 3b and Supplementary Fig. 13a) with D-HF demonstrating the highest elastic elongation value (Supplementary Fig. 13b). Surprisingly, while the drawn fibers showed higher elastic potential power on release of torsional stress than their non-rheologically controlled counterparts, the presence of CNTs was a preeminent factor, with the *V*_OC_ of HF (1.26 mV) comparable to D-GF (1.60 mV). The mismatch between *V*_OC_ and the shear modulus demonstrates that the effect of CNTs arises from the elastic region, where CNTs link the conductive network by a distance of 1–2 nm. The modulus of resilience (i.e., the area up to the yield point) is roughly equivalent to *V*_OC_, which alludes to the concept that CNTs transmit the power in the elastic regime (Supplementary Fig. 13c)^44–46^.

Furthermore, it can be seen that the interplay between CNTs and rGO plays a role in stress relaxation. By plotting *I*_SC_ profiles as a function of elapsed time while in transmission (Fig. 3g), HF stopped transmission after four revolutions, while D-HF slowly generated electricity after ten revolutions (Supplementary Fig. 13d, e). When the strain exceeds the yield point, the structure can be deformed irreversibly; however, the interplay between CNTs and rGO retarded the creep and relaxation processes as a result of increased energy storing capability. By taking advantage of drawing process during fiber spinning to improve short- and long-range order, D-HF transmitted the elastic potential energy into electrical energy 20 times that of HF and two times that of D-GF (HF = 0.004, D-GF = 0.04, D-HF = 0.08 MJ m^−3^, Supplementary Table 5).

### Comparison of mechanical strengths of D-HF

**Fig. 4:**
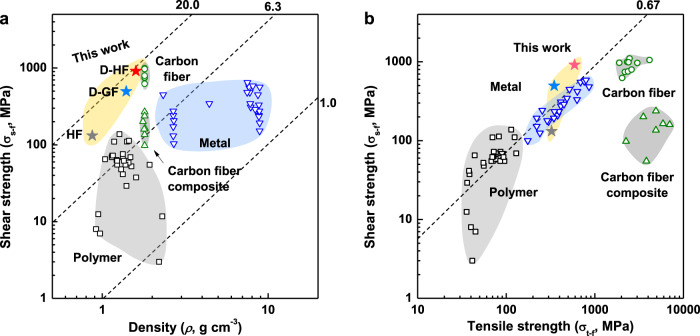
Comparison of mechanical strengths of the fibers. **a** Ashby plot of the shear failure strength (*σ*_s-f_) and density (*ρ*). The dotted lines denote the material indices for minimum mass design with limited strength corresponding to *σ*^1/2^/*ρ* values of 1.0 to 20.0 in units of MPa^1/2^/(g cm^−3^). They provide the slope of the family of parallel lines belonging to that index. All of the materials that lie on the same line perform equally well as a light, torsional shaft where those above the line perform better and those below perform worse. **b** Ashby plot of the shear and tensile strengths. The dotted line denotes the relationship between the shear strength and tensile strength based on the distortion-energy theory, machinery handbook, and Joerres’s modified Goodman relation.

The Ashby plot (Fig. 4a, D-HF red star) of the shear failure strength (*σ*_s-f_) and density (*ρ*) highlight the performance of these fibers versus metals (*σ*_s-f_ ~8.8× higher, *ρ* ~5.6× lower) and polymers (*σ*_s-f_ ~300× higher, comparable *ρ*), giving a strength/minimum-mass ratio^47^ of 19 MPa^1/2^ cm^3^ g^−1^, 2–14 times the values of metals, and 2–24 times those of polymers; 2–3 times higher than those of carbon fiber composites and similar with those of commercial carbon fibers^48^. A comprehensive table of density-normalized shear strength for structural materials is provided in the supplementary information (Supplementary Fig. 14 and Supplementary Table 6). Further, an Ashby plot of shear and tensile strength (Fig. 4b) is presented. The shear/tensile strength ratio of ductile materials are proportional to a specific constant (*C*)^49,50^ with tough materials typically following within a narrow range of shear/tensile strength ratios (*C* = 0.5–0.67). Most materials (including polymers and metals) follow this linear trend, with high strength, brittle materials (e.g. carbon fiber-based composite (*C* = 0.01–0.06), carbon fiber (*C* = 0.25–0.69)) showing significantly lower values. Strikingly, D-HF in this study shows high shear strength as well as tensile strength with a large constant (*C* = 1.5), attributed to the aligned and network structure enabling elastic flow of components in shear. The drawing and networking using CNT linker during fiber spinning manipulates the microstructure facilitating retention of energy throughout the duration of twisting along the axis.

## Discussion

In summary, high torsional strength nanocarbon fibers were created by coupling fluid dynamics, and mutual assembly of CNTs and (subsequently reduced) GO. The fibers under torsion demonstrate long-range plastic deformation with short-range elastic deformation, mediated by the structural configuration of constituents along the shear axis. The high torsional strength relies on a mobile hierarchical structure, which withstands shear stress efficiently across various size scales. During fiber spinning, the glassy-flow-like architecture is trapped in the solidifying fiber and may be exploited in the ‘flow’ of shear for the final material. Such flexible hierarchical systems are common in natural fibers exhibiting extraordinary torsional strengths. The quantitative extraction of elastic potential energy into electrical energy provides direct insights into how each constituent contributes to the torsional resistance by a series of rearrangements in response to twisting action, highlighting the importance of CNTs. The shear strength of our fibers outperformed other materials of comparable tensile performance and density. It should be noted that comparative mechanical properties are obtained from macroscale-isotropic materials.

## Methods

Fuller experimental details are provided in the Supplementary Information, including material sources, nanomaterial synthesis, purification, and dispersion.

### Rheological control in wet-spinning

Pre-prepared dispersion mixtures were extruded through a spinneret (inner diameter, 410 μm) at a fixed velocity (10 mL h^−1^) into the coagulation bath (5 wt% CaCl_2_ aqueous solution with 0.25 M NH_4_OH). The spun gel fibers in the bath were drawn faster than the extruding rate (0.024 m s^−1^). Then, the drawn gel fibers were conveyed continuously to a washing bath by a reel. To regulate the elastic asymmetry of the hybrid fibers, the fibers containing CNTs with a volume fraction of 0–0.3 were fabricated. The gel fibers were rinsed with water and dried at room temperature with a controlled humidity of 25%. The dried fibers were chemically reduced by a 30% HI solution at 80 °C for an hour. The rGO fibers were then washed with ethyl alcohol and dried at room temperature.

### Measurement of torsional mechanical properties

The torsional (shear) mechanical properties were measured using a rheometer (MCR 702 Twin-Drive, Anton Paar) with a specific accessary (solid rectangular fixture, SRF). For the measurement of the single-fiber mechanical properties, a square grid with a 25 × 15 mm hole in the center was designed. After the fiber was fixed in the middle of the grid, the fiber on the grid was safely loaded onto the rheometer. Then, the torsional mechanical property of the single fiber was measured after cutting off both sides of the grid. The investigations were performed at a torsional speed of 10 r.p.m. and a gauge length of 25 mm at room temperature and 25% humidity.

### In situ observation of electrical resistance change with torsional deformation

Fibers with a length of 25 mm were prepared. One end of the fiber was immovably fixed, and the other end was fixed to the motor shaft in parallel. By connecting both ends of the fiber to a multimeter (DMM 7510 1/2, Keithley), the electrical resistance of the fiber was measured in real time. The electrical resistance of the fiber was investigated as a function of the twisting number until breakage to the left at a rate of 10 r.p.m. The electrical resistance was monitored during the cycle test by repeatedly twisting the fiber 10 turns to the left and untwisting it 10 turns to the right at 10 r.p.m.

### Conversion of torsional elastic energy into electrical energy

Six coils with diameters of 25 mm were prepared by winding an enamel-coated copper wire (thickness: 1 mm) 50 times. The fixture of the coils was made from ABS using a 3D printer (3DISON, ROKIT). The magnet (2.34 g, 10 mm × 10 mm × 2 mm) was fixed at the end of the D-HF (7.04 × 10^−6^ g, 25 mm) located at the center of the six copper coils. The magnet was twisted by the motor shaft placed underneath. When the mechanically twisted fiber was released, the magnet made revolutions. While the fiber was rotating, the open-circuit voltage and short-circuit current were measured in real time.

## Supplementary information

Supplementary Information

Supplementary Movie 1

Description of Additional Supplementary Files

## Data Availability

The datasets generated during and/or analyzed during the current study are available from the corresponding author on reasonable request.
