# Supplementary material for: Carbon nanotube-reduced graphene oxide fiber with high torsional strength from rheological hierarchy control
Source: Nat Commun. 2021 Jan 15;12:396. doi: 10.1038/s41467-020-20518-0 (PMC7810860; doi:10.1038/s41467-020-20518-0)
Supplement: Supplementary file 1 — Supplementary Information [file 41467_2020_20518_MOESM1_ESM.pdf]

Supplementary Information

Carbon Nanotube-Reduced Graphene Oxide Fiber with High Torsional Strength from Rheological Hierarchy Control

Eom et al.

Materials and Methods

1. Materials

Graphite powder was purchased from Asbury Carbons (grade 2012). CNTs were purchased from RND Korea (KRU4200). Calcium chloride ($CaCl_2$) and ethyl alcohol were purchased from Samchun Chemical Co. Ltd. Chemicals, including hydroiodic acid (HI), sulfuric acid 95% (H_2SO_4), nitric acid 70% (HNO_3), and ammonia solution (NH_4OH), were purchased from Junsei Chemical Co., Ltd. Deionized water (DIW) was obtained using a water purification system purchased from Millipore (Direct Q3). The epoxy adhesive used to fix the fibers was purchased from ITW Performance Polymers (Devcon).

2. Preparation and characterization of GO and CNT dispersion

Graphite (10 g) and concentrated H_2SO_4 (380 mL) were stirred together in a double-jacket reactor and cooled to 5 ^oC using the circulator. Potassium permanganate (50 g) was slowly added to the mixture with the temperature kept below 12 ^oC. Then, the reactor temperature was elevated to 35 ^oC and the mixture was stirred for 12 h. The mixture was cooled to 5 ^oC again, and deionized water (2500 mL) was added slowly, ensuring the temperature was kept below 10 ^oC. Next, H_2O_2 (30 mL) was poured into the mixture and the mixture became orange brown. The synthesized GO was filtered and washed with HCl (1M, 2000 mL) over a cellulose membrane (Whatman filter paper, 2.5-μm pore size, Millipore Inc., Billerica, MA). The obtained GO cakes were dried in the dark at room temperature for a week. Then, the GO was redispersed in acetone (1000 mL) and filtered and washed with acetone (2000 mL) over the cellulose membrane. The GO was dried at room temperature for 72 hours. The obtained 2.0-g GO powder was dispersed in 1L DIW by bath sonication at 200W for 30 min. The dispersion was centrifuged at 4000 rpm for 1 hour, and only the GO in the supernatant was obtained. Then, the supernatant was centrifuged at 8000 rpm for 1 hour and the extremely small debris was removed by dispensing the supernatant. The typical concentration of the final stock GO dispersion was approximately 1.0 wt%. To improve the dispersibility of CNTs in aqueous solution, CNTs were oxidized using strong acid solution. A total of 200 mg of CNT powder was poured into 400 mL of H_2SO_4/HNO_3 3:1 v/v solution, oxidized for 6 hours at 65 ^oC, and cooled at room temperature overnight. Before washing the CNTs using a filtration system (0.45-μm

PVDF membrane, Millipore), the strong acid solution was diluted with 2L DIW to prevent damaging the membrane. The CNTs were washed until the pH was approximately 7. Then, they were centrifuged at 15000 rpm for 30 min twice and the precipitate was discarded to remove impurities, such as unmodified CNT. Finally, the homogenous CNT dispersion was obtained.

3. Characterizations

The optical birefringence of the CNT/GO dispersion was characterized with polarized optical microscopy (POM, ECLIPSE LV100N POL., Nikon). A droplet of the dispersion on a glass substrate was observed using a halogen lamp, and the polarizer was fixed to the bottom of the condenser holder. The dynamic rheological properties were measured with a MARS III rheometer (Thermo Scientific) in oscillatory and steady modes. In the oscillatory mode, the condition was 5% strain at 20 ^{o}C (parallel plate geometry, 20 mm in diameter, complementarity determining region of 0.1 radians, and a gap of 0.5 mm). The GO sheets and CNTs were observed via AFM (XE-70, Park Systems) in tapping mode. The measured data were processed using data-processing and analysis software (XEI, Park Systems). The SAXS analysis was studied with reduced graphene oxide fiber at the Pohang Accelerator Laboratory (PAL) on a 4C beamline with an x-ray beam wavelength of 0.675 Å at a sample-to-detector distance of 1 m. To investigate the inter-layer distance of CNT-intercalated GO sheets and crystallite size, XRD (mini Flex 600, Rigaku) was carried out using Cu Kα radiation (λ = 1.5418 Å). The mass of the fibers was measured with an ultra-microbalance (maximum capacity: 2.1 g, readability: 0.1 μg, Mettler Toledo), and the density and denier were calculated. At least five fibers of 7 cm were measured to ensure accuracy. The morphology of the fibers was characterized using SEM (Hitachi S4800) with the conditions of 15 kV and 10 μA without Pt sputtering. To directly visualize the structural defects inside of the fibers, X-ray nano-imaging (XNI) was employed using the synchrotron analysis. CNT was analyzed on JEM-F200 TEM installed at the National Center for Inter-university Research Facilities (NCIRF) at Seoul University. Through Cryo-TEM analysis, the cut slide in the vertical and horizontal directions of the fiber axis were observed. To observe clear cross-section in cryo-TEM, all fibers were immersed into molds with epoxy resin (Low viscosity embedding media Spurr's kit). The ingredients are

vinyl cyclohexene dioxide (ERL 4221), diglycidyl ether polypropylene glycol (DER 736), nonenyl succinic anhydride (NSA) and dimethylamino ethanol (DMAE). After curing the epoxy at 70 °C for 8 h, the samples were cut into small slides using a Leica UC7 Microtome cutting machine. The cut slide was manually placed directly on the TEM Cu mesh grid for observed. The twisted fiber sample was prepared by the following method. One end of the fiber was fixed with adhesive to prevent rotation, and the other end was twisted at 10 rpm for 66% of the breaking twist number and then fixed with adhesive. During the preparation, longitudinal shrinkage was restricted. The tensile mechanical properties of the fibers were investigated using a universal testing machine (5966, Instron) equipped with a 10-N load cell. Ten fibers were prepared for each sample. The test was operated at a 2.5-mm min^{-1} crosshead speed at a gauge length of 25 mm by referring to the tensile measurement of single ultrafine fibers. The torsional modulus is obtained from torsion data in Fig. 3b, as an average calculated from total torsional force as a function of total cross-sectional area (modelled as rectangular from TEM cryo-fracture min/max orthogonal diameters to provide lower bound values), calculated using non-linear curve fitting in Origin Pro software. Notably, D-HF at high strain (> ~12%) deviated from a linear proportionality and established an exponential increase in torsional stress with increasing strain. The calculated torsional modulus indicates that the sum of the modulus of the cross section.

The electrical conductivity of the prepared fibers was measured with a potentiostat (Ivium-n-stat, Ivium Technologies). Linear voltammetry was obtained at a scan rate of 50 mV s^{-1} at a voltage range of -0.8 to 0.8 V. The electrical conductivity was obtained by the calculation using the measured electrical resistance, the cross-sectional area observed through SEM, and the resistance measurement length. The fiber was observed by optical microscopy during twisting. One end of the fiber was fixed with epoxy adhesive, and the other end was fixed to the motor shaft and rotated.

4. Calculation of degree of orientation from SAXS and WAXS

Herman's orientation factor (*f*), which is given by

$$f = \frac{3\langle \cos^2 \theta \rangle - 1}{2} \quad (1)$$

where θ is the angle at the azimuthal plot and $\langle \cos^2 \theta \rangle$ is the average value of the square of the cosine of the angle θ. Assuming the rotational symmetry of the fiber axis, the following equation is obtained.

$$\left\langle \cos^2 \theta \right\rangle = \frac{\int_0^{\pi/2} I(\theta)\cos^2 \theta \sin\theta d\theta}{\int_0^{\pi/2} I(\theta)\sin\theta d\theta} \quad (2)$$

where I(θ) is the intensity at θ in the azimuthal scan collected from the SAXS data (Figs. 2a–d).

5. Calculation of inter-layer distance and crystallite size from XRD.

The inter-layer distance was obtained by calculating the XRD data using Bragg's law.

$$d = \frac{\lambda}{2\sin\theta} \quad (3)$$

where d is the inter-layer distance, λ is the wavelength (1.54 Å) of the X-rays, and θ is the diffraction angle. The crystallite size was also calculated with Scherrer's equation in the XRD data.

$$L_c = \frac{K \cdot \lambda}{B\cos\theta} \quad (4)$$

where L_c is the average crystallite size, K is Scherrer's constant (0.89), λ is the X-ray wavelength (1.54 Å), B is the FWHM, and θ is the diffraction angle.

6. Calculation of the porosity of the fibers.

The porosity of fibers was derived from the structural parameters using the following equation:

$$Porosity(\%) = 1 - \frac{\rho_f d_{002}}{\rho_f d_g} \quad (5)$$

where ρ_f is the fiber density, ρ_g is the graphite density (=2.26 g/cm^3) and d_g = 0.3354 nm for graphite crystals.

7. Calculation of stored and emitted kinetic energy.

The initial acceleration generated by the magnetic paddle was 57.9 rad s^{-2} (3315.7° s^{-2}), and the inertia of the magnet paddle (I) was 4.88 x 10^{-9} kg m^2 according to

$$I = \frac{ml^2}{12} \tag{6}$$

where m is the mass of the magnet paddle and l is the diameter of the magnet paddle. Therefore, the maximum specific torque (τ) was calculated as 40.08 N · m kg^{-1} according to $\tau = I\alpha$. In addition, the kinetic energy generated in the magnet paddle ($I\omega^2/2$), normalized by the 0.33 s needed to accelerate the initially stationary paddle to an angular velocity of ω = 19.07 rad s^{-1}, provided a peak power output of 382.11 W kg^{-1}.

Supplementary Text

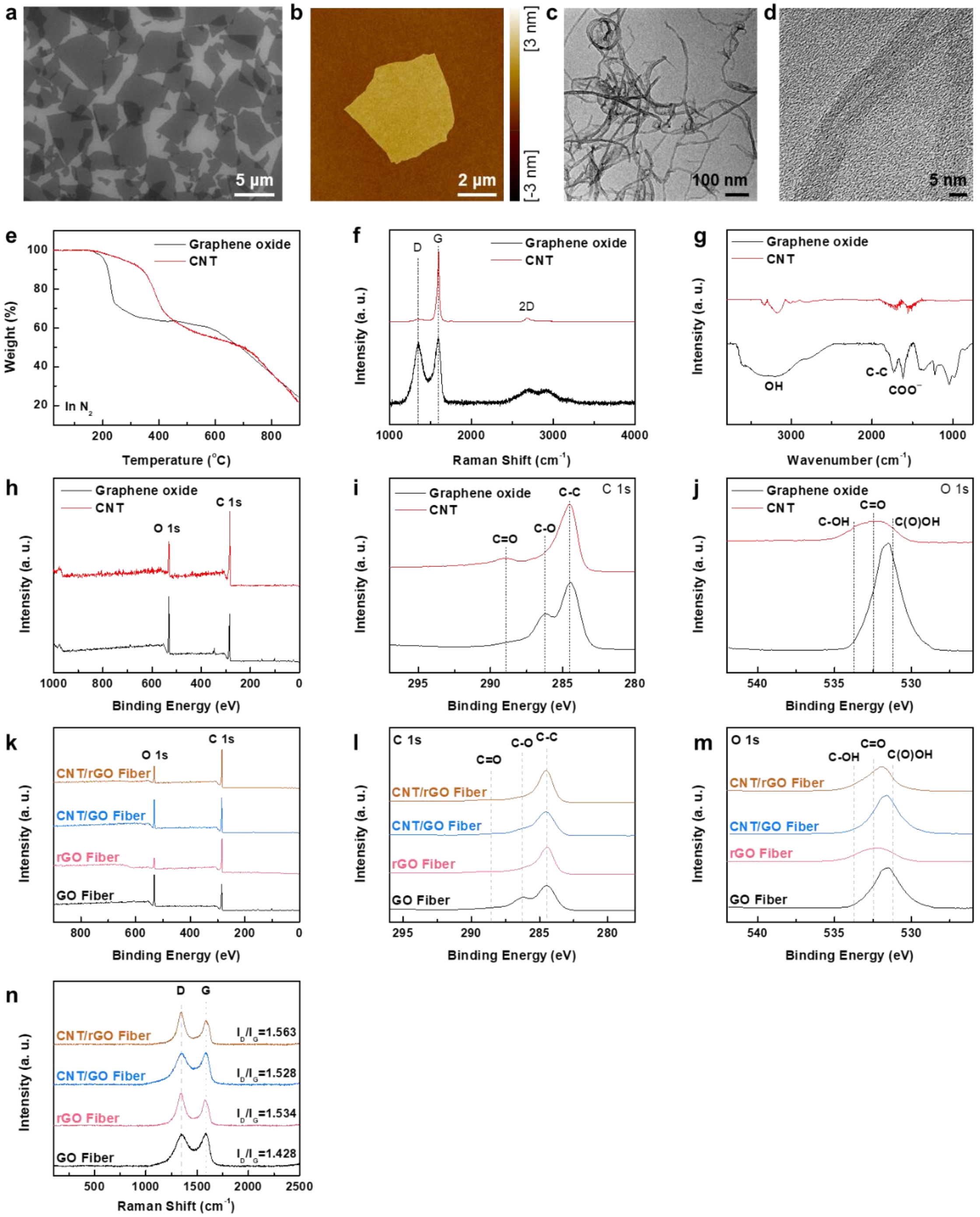

Supplementary Figure 1. Characterization of GO and CNT. **a** SEM and **b** AFM images of GO. **c** TEM and **d** HR-TEM images of CNTs. **e** TGA thermograms, **f** Raman spectroscopy, **g** FTIR spectra, **h** XPS survey, **i** XPS C 1s spectra, and **j** XPS O 1s spectra of GO and CNT. **k-m** XPS and **n** Raman spectra of GO, rGO, CNT/GO and CNT/rGO fiber.

Note: Characterization of the GO and CNT

Full characterization of graphene oxide (GO) and oxidized carbon nanotubes (CNT) were performed including scanning electron microscope (SEM), atomic force microscopy (AFM), transmission electron microscopy (TEM), thermogravimetric analysis (TGA), Raman spectroscopy, Fourier transform-infrared spectroscopy (FT-IR) and X-ray photoelectron spectroscopy (XPS). The used GO sheets are monolayer and have a uniform size distribution (**Supplementary Figure 1a, b**). And the used CNT has a thickness of 8.29 ± 0.88 nm (**Supplementary Figure 1c, d**). To manufacture the homogenous GO and CNT dispersion, both of GO and CNT has chemical functional groups on their surface. In **Supplementary Figure 1e**, the dramatic decrease of GO and CNT at 190 and 320 °C, respectively, presented the existence of the functional groups. The D and G peaks in Raman spectra showed the functional groups were combined with sp^2 carbon structure (**Supplementary Figure 1f**). They were mainly oxygen functional groups including hydroxyl, carboxylate, and epoxide (**Supplementary Figure 1g**). The fact was also double-checked by XPS analysis in **Supplementary Figure 1h-j**. The presence of -OH, -OOH and COO- is confirmed in XPS C 1s and O 1s.

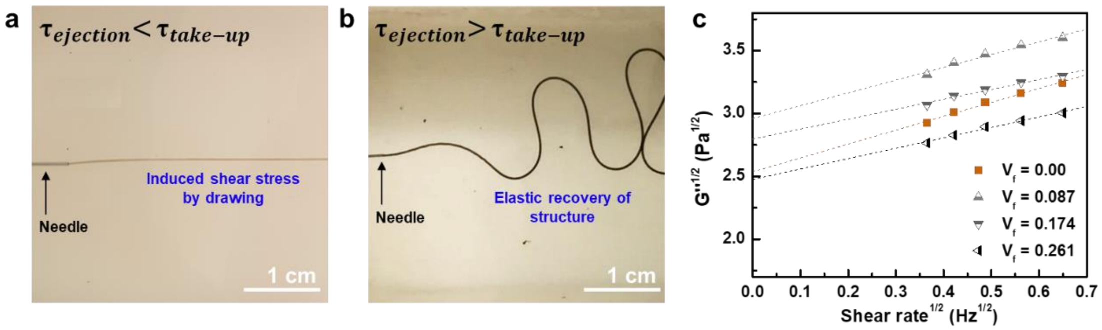

Supplementary Figure 2. a, b Image of the extrusion with and without rheological control via take-up. **c** Calculated yield stress based on Casson's plot. The trend lines based on experimental data are extrapolated to the y-axis, which is the square root of the loss modulus.

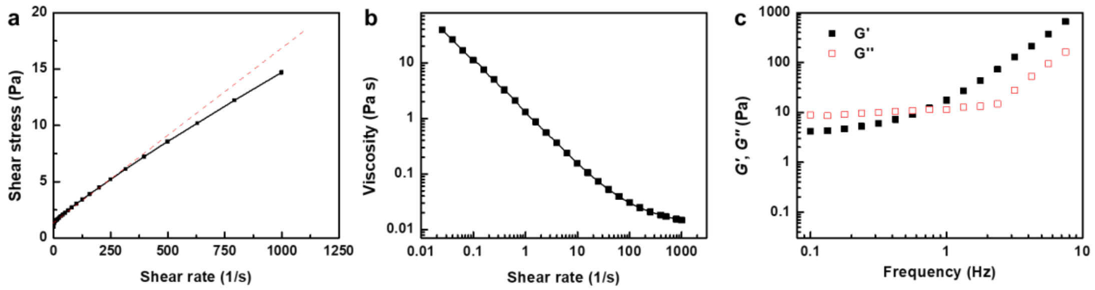

Supplementary Figure 3. Rheological properties of the GO solution (5 mg mL^{-1}): **a** shear stress and **b** viscosity as a function of shear rate. **c** G' and G'' of GO dispersion as a function of shear frequency.

Note: Calculation of stress induced by ejection rate and take-up rate using Navier-Strokes equation

In order to align coagulated lyotropic liquid crystal dispersions, a shear stress above the yield stress is required. The applied shear stress to a dispersion is induced by the ejection and take-up rates. We selected a model system suitable for GO LC and used microfluidics to calculate the ejection and take-up rates to generate a shear stress above the yield stress. (**Supplementary Figure 2a, b**)

1. Selection of rheological model system and equation

Assuming that the rheological behavior of the GO dispersion is accounted for in the form of Stokes flow (incompressible Newtonian fluid) with a normal directional flow without a nonlinear term, an approximate solution of the Navier-Stokes equation of motion is used. Because although the GO LC dispersion is a Bingham pseudoplastic fluid, it has a nearly constant viscosity at high shear rates. As supporting evidences, the measured rheological results of 5 mg mL^{-1} GO LC dispersion are presented in **Supplementary Figure 3**. The GO dispersion showed shear thinning behavior along with a yield stress (1.4 Pa) and the fitted red line represents the shear thinning properties of the fluid. In **Supplementary Figure 3a**, at a high shear rate, the GO dispersion, like a Newtonian fluid, exhibited a nearly constant viscosity. We have previously observed that the GO dispersion under flow in the nozzle demonstrated a parabolic velocity profile, indicating a laminar flow,[18] in spite of despite the dispersion's non-Newtonian nature (**Supplementary Figure 3b**). Based on our previous work, we experimentally know that GO gel fiber is spun uniformly when the dispersion is extruded at a shear rate of 411.5 1/s (corresponding to 10 mL h^{-1}) by using a needle (diameter: 410 µm) in this study (**Supplementary Figure 3b**). At this high shear

rate, the GO dispersion reveals a low constant dynamic viscosity of 0.018 Pa s. The shear rate $\dot{\gamma}$ is calculated by using the following equation.

$$\dot{\gamma} = \frac{4Q}{\pi r^3} \tag{7}$$

where Q is the volumetric flow rate and r is the radius of the needle

2. Calculation of mean shear stress from ejection rate

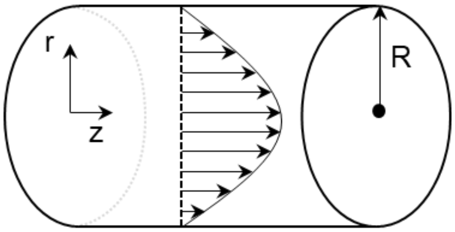

Circular tube due to pressure difference.

Assuming that the incompressible Newtonian fluid flows in one direction in steady state, the velocity distribution of the fluid flowing through a circular tube due to a pressure difference P can be expressed as:

$$u = \frac{G}{4\mu}(-r^2 + C_1 \ln r + C_2) \tag{8}$$

where u is velocity distribution, G pressure gradient, μ dynamic viscosity, r distance from the center, and C_1 and C_2 constants that are determined by using the following appropriate boundary condition.

r = 0, u is finite

r = R, u = 0

thus,

$$u = \frac{GR^2}{4\mu}\left[1 - \left(\frac{r}{R}\right)^2\right] \tag{9}$$

This is expressed as a dimensionless variable as follows.

$$\tilde{u} = 2(1 - \tilde{r}^2);\ \tilde{u} = \frac{u}{GR^2/8\mu},\ \tilde{r} = \frac{r}{R} \tag{10}$$

Because the GO dispersion with shear stress greater than the yield stress showed limited structural modification, such as an alignment of the lamellar planes, but no viscous

components, if the mean velocity of the fluid $\langle u \rangle = u_c$, then the maximum velocity is $u_{max} = 2u_c$. Since the velocity distribution has been determined, the volumetric flow rate Q is

$$Q = \int_0^{2\pi} \int_0^R urdrd\theta = \frac{\pi G R^4}{8\mu} \tag{11}$$

The shear stress is now determined as follows.

$$\tau_{rz} = \tau_{zr} = \mu \frac{\partial u}{\partial r} = -\frac{G}{2} r \tag{12}$$

Since the τ_{rz} is a function of G, and G is a function of the velocity Q, it can be expressed as a function of Q (τ_{rz}). Further the mean shear stress $\langle \tau_{rz} \rangle = \tau_{max}/2$ when R = r.

$$Q = -\frac{\langle \tau_{rz} \rangle \pi R^3}{2\mu} \tag{13}$$

where μ is experimentally measured as a 0.018 Pa s and R is 205 μm. Thus, the mean shear stress by ejection ($\tau_{ejection}$) induced by the ejection rate ($\gamma_{ejection}$) has the following equation.

$$\tau_{ejection} = \frac{2\mu\gamma_{ejection}}{\pi R^3} \tag{14}$$

Considering the experimental yield stress of the prepared dispersion, a mean shear stress of at least 1.93 Pa is necessary to produce viscous flow. According to the calculation performed based on the model system, the dispersion should be ejected at a rate above 10.99 mm s^{-1} to apply a shear stress above 1.93 Pa. Reflecting the theoretical calculation, in the experiment, the dispersion was ejected at a rate of 21.05 mm s^{-1} which is higher than the minimum required ejection rate (> 10.99 mm s^{-1}). The experimental ejection rate and the applied shear stress (3.70 Pa) are about twice the minimum required ejection rate and stress, respectively. It is considered that this difference is due to a gradient of the shear stress that depends on the position inside the needle, the resistance offered by the coagulating solution during ejection, the hydraulic pressure corresponding to the height of the coagulating solution, and the rate at which the cations in the coagulating solution infiltrate backward into the needle.

3. Calculation of mean stretching stress from take-up rate

The fiber were stretched by take-up roller immediately after ejected outside for the nozzle. The difference between the flow rate of fluid in nozzle and take-up rate accomplishes the fiber to stretch. During the stretch, elongational tension is applied to the fiber and the tensile stress has the following equation.

$$\sigma(t) = F(t) \times A(t) \tag{15}$$

where *F(t)* is tensile force and *A(t)* is cross-sectional area at time t. However, in this study, GO gel is too weak to measure. Instead, based on our previous observations regarding the ideal flow exhibited by the solution (**Supplementary Figure 3b**), we treat the GO solution as an incompressible Newtonian fluid, and assume both elongational and shear flows obey Newton's law of viscosity. Thus, the applied stress by take-up rate can be predicted by Navier-Strokes equation in shear. The stress ($\tau_{take\text{-}up}$) induced by the take-up rate ($\gamma_{take\text{-}up}$) at a fixed $\gamma_{ejection}$ has the following equation.

$$\tau_{take-up} = \frac{2\mu\gamma_{ejection}}{\pi\left(\sqrt{\frac{Q}{\pi\gamma_{take-up}}}\right)^3} \tag{16}$$

For maintaining or improving the alignment of the GO and CNT assembled structure fabricated by using $\tau_{ejection}$, the $\tau_{take\text{-}up}$ and $\gamma_{take\text{-}up}$ should be higher than the experimental $\tau_{ejection}$ (3.7 Pa) and $\gamma_{ejection}$ (21.05 mm sec^{-1}), respectively. The experimental $\gamma_{take\text{-}up}$ was 23.55 mm s^{-1}, which induced a $\tau_{take\text{-}up}$ of 4.37 Pa.

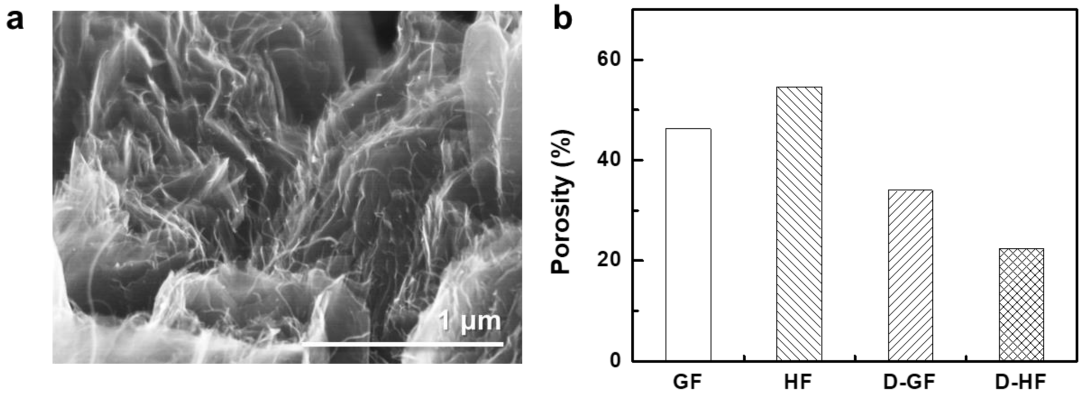

Supplementary Figure 4. a Magnified cross-sectional image of D-HF. **b** The porosity of GF, HF, D-GF and D-HF. The interaction of CNT with GO is presented. It is noted that the increased density of fiber is implicated in strong interaction between rGO and CNTs. In the broken cross section of fiber, the well distributed CNT is observed (Supplementary Figure 4a).

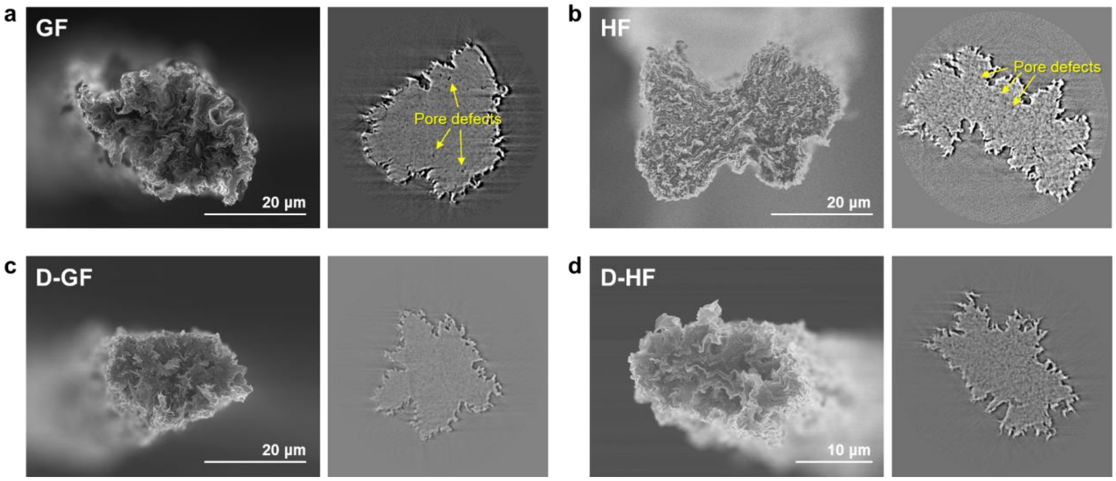

Supplementary Figure 5. The cross-sectional SEM and XNI of **a** GF, **b** HF, **c** D-GF and **d** D-HF. Structure analysis of fiber to see structural defects, such as voids, using X-ray nano imaging technology. (Experiments were carried out at Pohang Accelerator Laboratory (PAL, 7C))

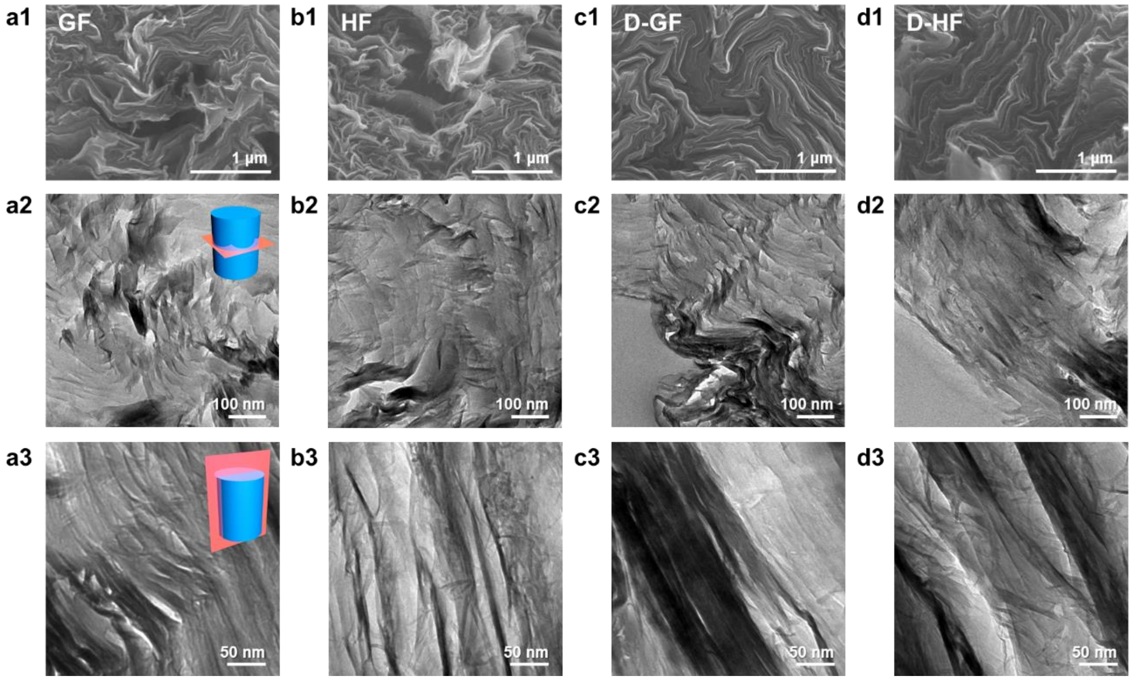

Supplementary Figure 6. Cross-sectional cryo-SEM of **a1** GF, **b1** HF, **c1** D-GF, and **d1** D-HF. TEM images of the cutting surface in the vertical and horizontal directions of the fiber axis: **a2,3** GF, **b2,3** HF, **c2,3** D-GF, and **d2,3** D-HF. From the differences of the images in the vertical direction, the cross-section of the fiber shows that the pore defects are reduced by alignment and the gap between the sheets is narrowed to form a dense structure. While the addition of CNTs without drawing process results in pore defects and cracks, CNT addition with drawing process contribute to a more compact structure of fibers. The dispersibility and degree of alignment of added CNTs were observed from the cutting surface in the horizontal direction. While the CNTs without drawing process are aggregated and not oriented to the fiber axis, the CNTs with drawing process are homogenously dispersed and oriented to the fiber axis.

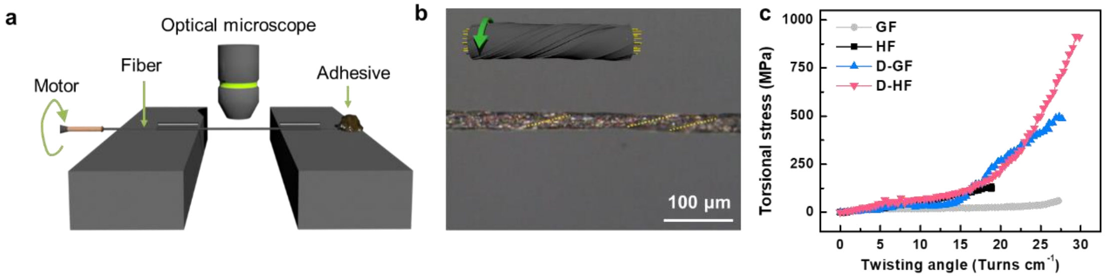

Supplementary Figure 7. a Schematic illustration of the twisting process. **b** Morphology of twisting D-HF observed by an optical microscope. **c** Torsional stress curve as a function of twisting angle.

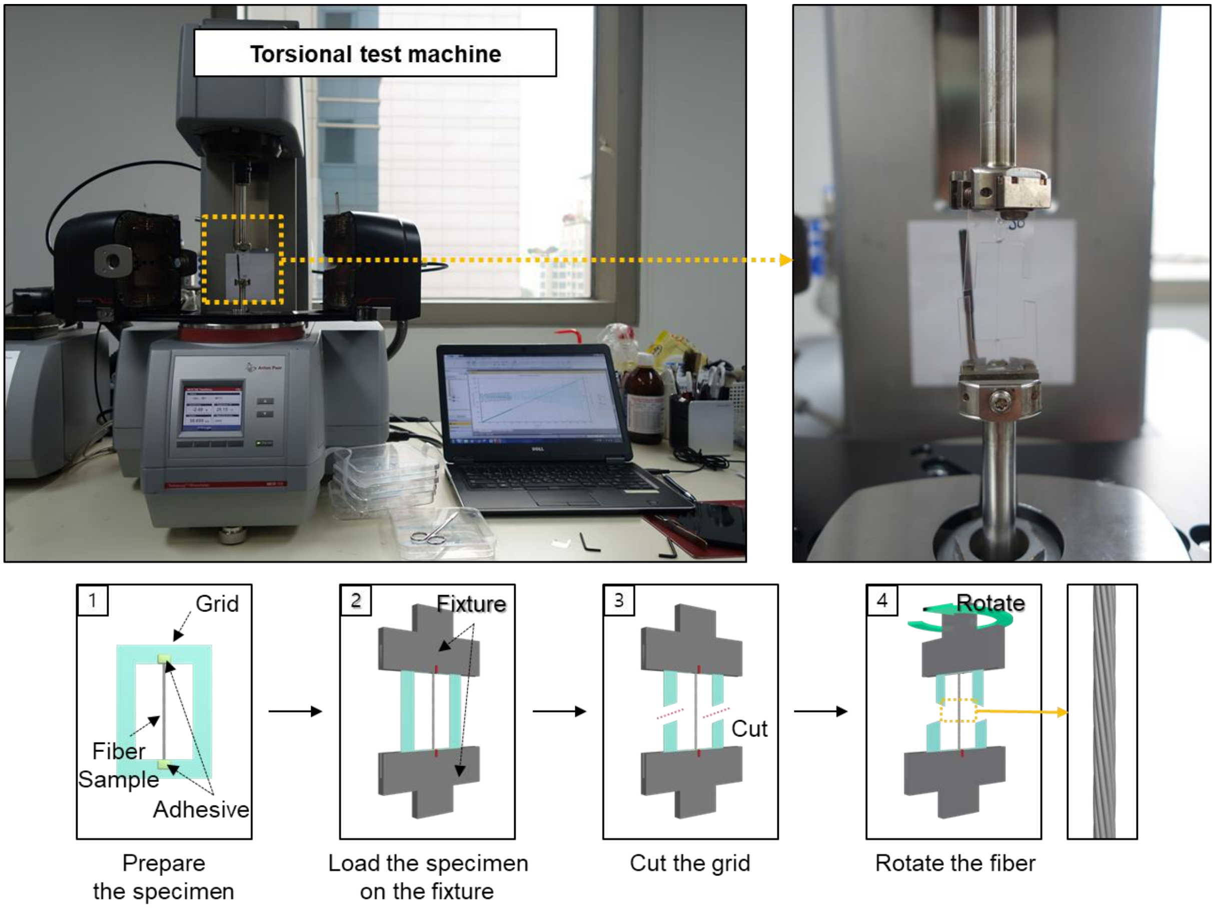

Supplementary Figure 8. The torsional stress-strain curves were measured by using a rheometer (MCR 702 Twin-Drive, Anton Paar) with a specific accessory (solid rectangular fixture, SRF). Step 1: Prepare the single fiber sample loaded on the grid. Step 2: Load the specimen on the fixture (SRF). Step 3: Before starting the test, cut off the pillars on both sides. Step 4: Test the fiber.

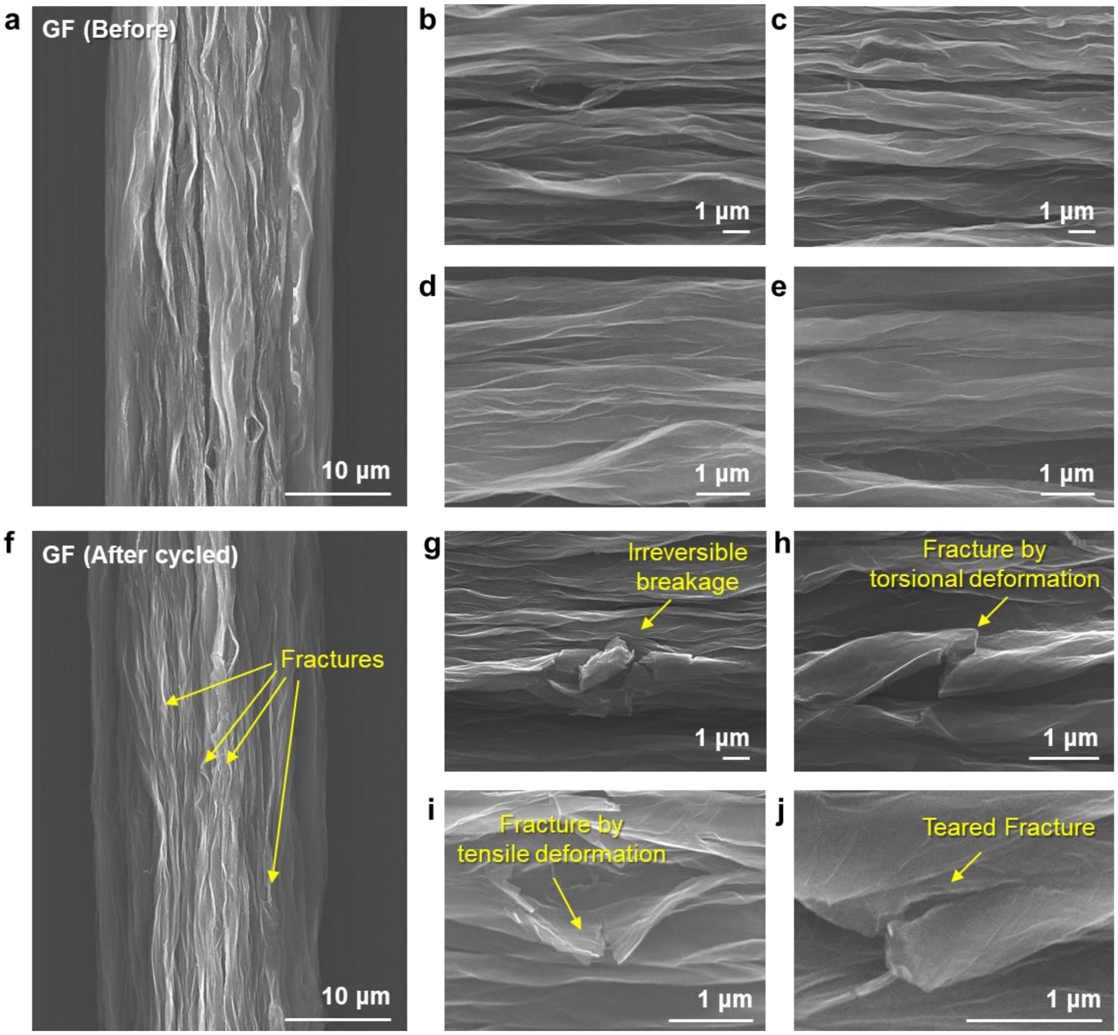

Supplementary Figure 9. Morphologic alteration of GF after twisting experiments in Fig. 3d.

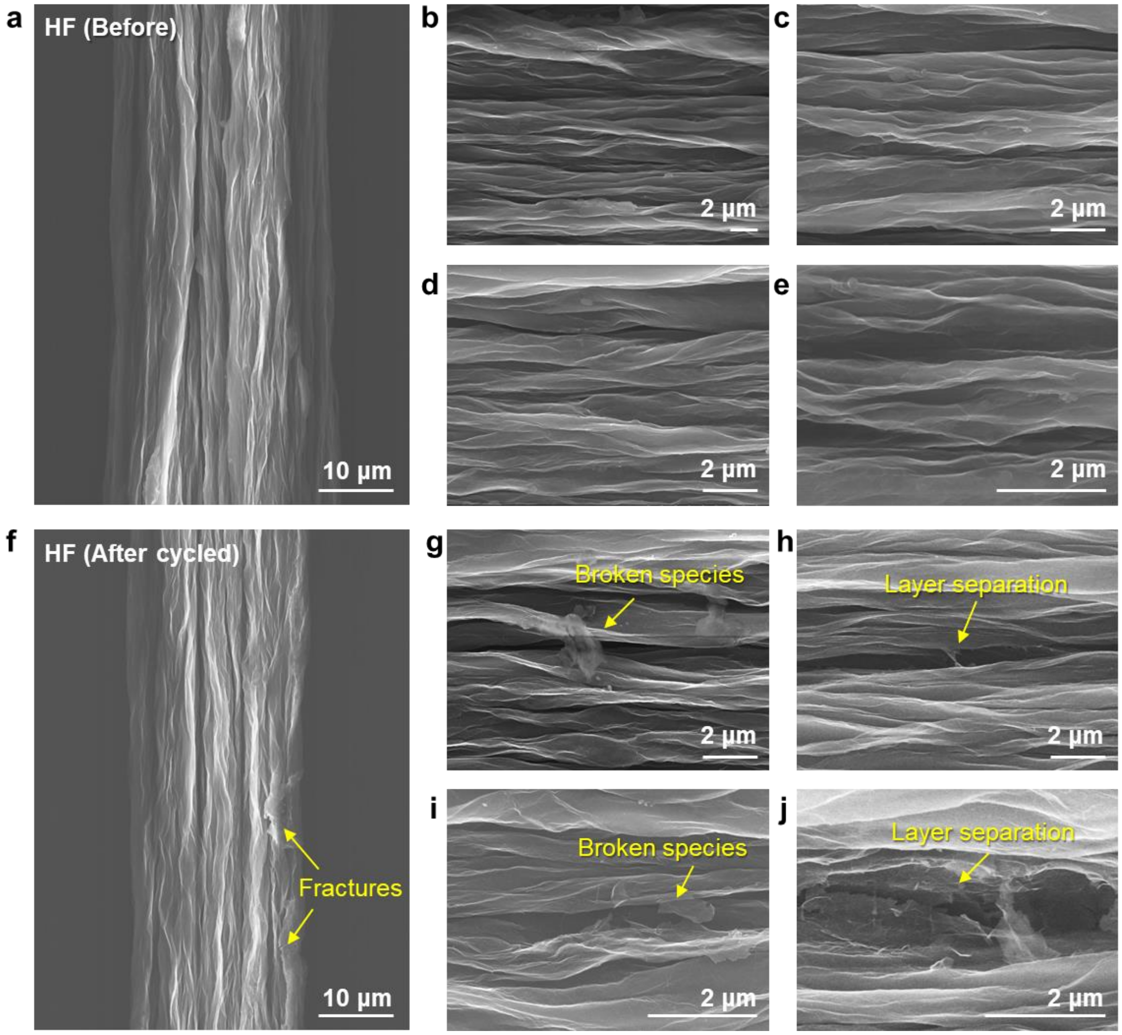

Supplementary Figure 10. Morphologic alteration of HF after twisting experiments in Fig. 3d.

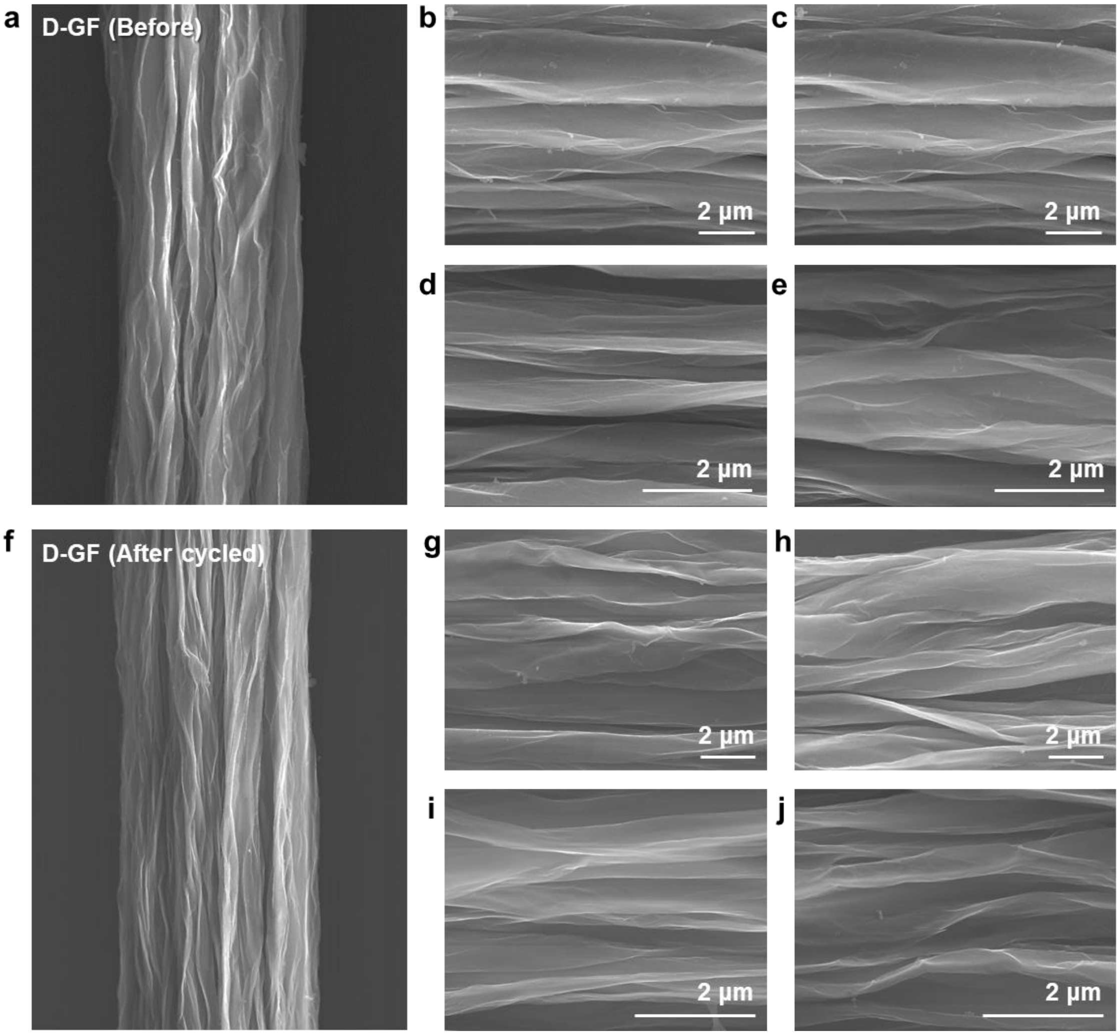

Supplementary Figure 11. Morphologic alteration of D-GF after twisting experiments in Fig. 3d.

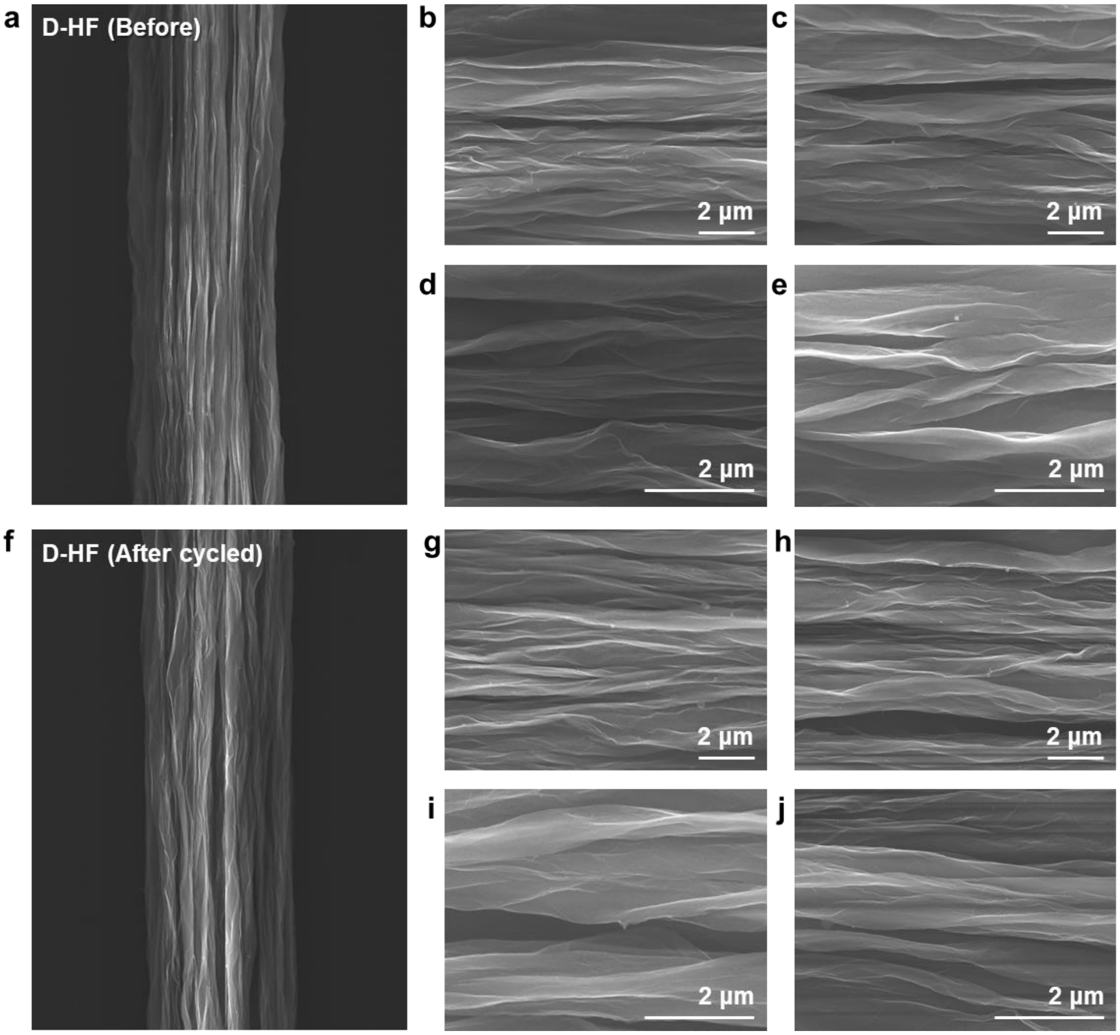

Supplementary Figure 12. Morphologic alteration of D-GF after twisting experiments in Fig. 3d.

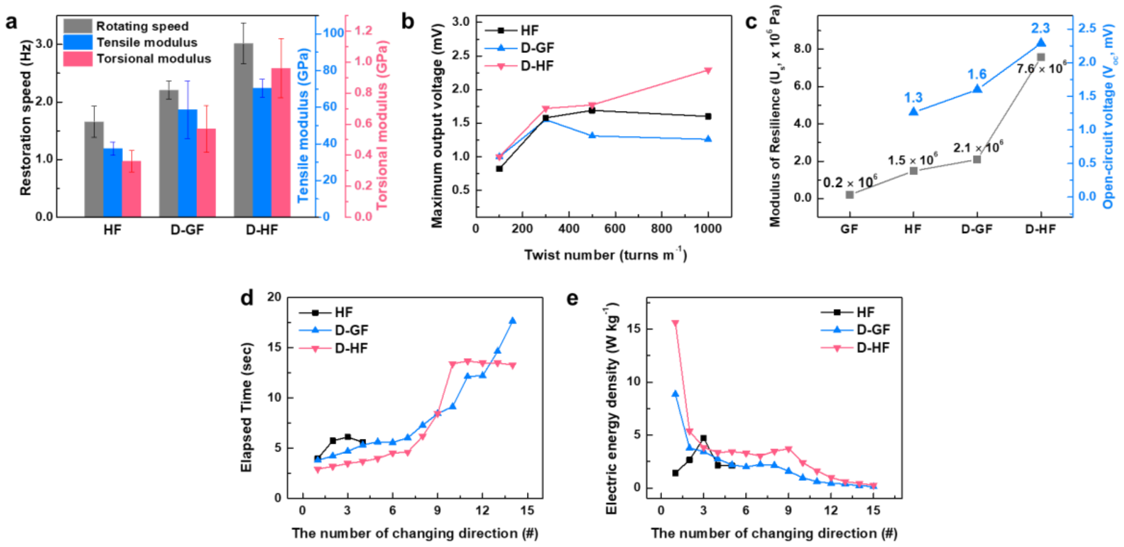

Supplementary Figure 13. a Modulus of resilience and open-circuit voltage of fibers. **b** Maximum open circuit voltage as a function of the twist number. **c** Summarized results of the rotating speed, tensile modulus, and torsional modulus for different fibers. **d** Elapsed time and **e** electric energy density depending on the number of changing directions of released fibers after twisting.

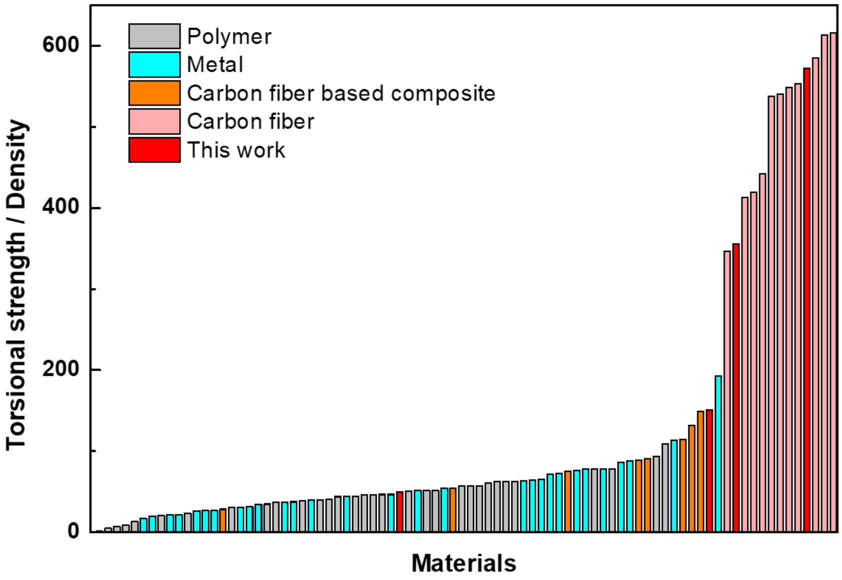

Supplementary Figure 14. The torsional strength values normalized by the density of GF, HF, D-GF and D-HF is compared with current conventional materials. The torsional strength values normalized by the density of GF, HF, D-GF and D-HF is compared with current conventional materials. Most metals and polymers show values below 100, and several metals (Aluminum and silicon) have values above 100. Although the value of rGO fiber without drawing process (GF) showed 50, the value of D-HF was 573, which is 5 times higher than metal and similar with the values of carbon fiber.

Supplementary Table 1. Fitted linear functions of Casson's model

**Equation**	**Loss modulus$^{1/2}$ = a + b × Shear rate$^{1/2}$**		
**Volume fraction (V_f)**	**Intercept (a)**	**Slope (b)**	**Yield Stress (At Shear rate = 0, Pa)**
0.00	2.53637	1.10381	6.43
0.087	2.96096	1.01369	8.77
0.174	2.79691	0.78838	7.82
0.261	2.47593	0.827	6.13

Note: Calculation of yield stress using Casson's equation

The non-Newtonian properties of a fluid are calculated by using Casson's model. Since the properties of GO and GO/CNT dispersions were measured in oscillatory mode, the loss modulus, which depends on frequency represents the yield stress, based on Cox-Merz rule. The yield stress is calculated by using the equation below.

$$G''^{1/2} = \tau y^{1/2} + (\mu\gamma)^{1/2} \quad (17)$$

where G″ is the shear stress, τ_y the yield stress, μ the shear viscosity and γ the shear rate. The loss modulus values in low shear rate ranges were used to extrapolate to determine the y-axis find intercept.

Supplementary Table 2. DLVO interaction model-fitted parameters of sheets with nanotube dispersions

**Volume fraction (V_f)**	**s_0 (nm)**	**r_0 (nm)**	**α**	**G' (Pa)**	**ϵ_{yield} (%)**	**σ_{yield} (Pa)**
0.00	0.806	2.21	5.29×10^{-16}	11.09	6.04×10^{-1}	6.70
0.087	0.956	2.36	7.44×10^{-16}	13.40	6.37×10^{-1}	8.54
0.174	0.974	2.37	7.72×10^{-16}	11.46	6.41×10^{-1}	7.34
0.261	1.045	2.44	8.95×10^{-16}	9.03	6.53×10^{-1}	5.90

Note: Calculation of the yield stress using the Derjaguin-Landau-Verwey-Overbeek (DLVO) interaction model

The rheological properties of dispersion were investigated based on DLVO interactions. The lateral shear motion is shown to be elastic up to a distance of y_{max}. If s_0 and r_0 are the closest center-to-center and surface-to-surface distances, respectively, between two particles,

$$y_{max} \propto (1-0.5\alpha\zeta^2)(s_0 r_0)^{1/2} \tag{18}$$

where ζ is the zeta potential of the particles and α is a defined constant. s_0 and r_0 referred to the inter-layer distance between GO sheets and the thickness of single sheet. Meanwhile, α is defined as

$$\alpha \equiv \left(2\pi\varepsilon_0\varepsilon_w k \frac{e^{-ks_0}}{1+e^{-ks_0}} \right) \Bigg/ \left(\frac{A}{12{s_0}^2} \right) \tag{19}$$

where ε_0 and ε_w are the static dielectric constants of the particle and water, which are 8.85 × 10^{-12} C^2 J^{-1} m^{-1} and 78.49, respectively. k is the inverse of the screening length in the Debye-Hückel approximation; 1.4 × 10^9 m^{-1}. A is the Hamaker constant with water as the medium, which can be calculated by using the Tabor-Winterton approximation, 4.5 × 10^{-20} J. Returning to y_{max}, the yield strain can be approximated from y_{max} to

$$Strain_{yield} = \frac{y_{max}}{r_0} \propto (1-0.5\alpha\zeta^2)\left(\frac{s_0}{r_0}\right)^{1/2} \tag{20}$$

The shear modulus of the network, G′, can be deduced by combining the elastic constant of the lateral movement with the existing elastic theory of a particle network. The yield stress can be approximated as follows.

$$\sigma yield \approx G' Strain_{yield} \propto (1-0.5\alpha\zeta^2)\frac{A}{24{s_0}^{3/2}}\frac{1}{R^{d-3/2}} \tag{21}$$

However, since the model we referred to is based on nanospheres, which do not reflect the 2D nature of GO, the experimental G' value is substituted. Finally, the calculated σ_{yield} is presented in **Supplementary Table 2**.

Supplementary Table 3. Yield strengths of the GO-only and GO/CNT solutions (experiments and model calculations)

	Volume fraction (V_f)	Experimental (Pa)	Casson's model (Pa)	DLVO model (Pa)
GO	0.00	1.63 ± 0.01	6.43	6.70
GO and CNTs	0.087	1.931 ± 0.012	8.77	8.54
	0.174	1.633 ± 0.014	7.82	7.34
	0.261	1.322 ± 0.012	6.13	5.90

Supplementary Table 4. Tensile and shear properties and electrical conductivities of the fibers in this study.

Sample	Specific density (g cm^{-3})	Tensile properties		Shear properties		Electrical conductivity (S cm^{-1})
		Ultimate strength (MPa)	Young's modulus (GPa)	Ultimate strength (MPa)	Shear modulus (GPa)	
GF	1.06 ± 0.10	229 ± 40	28 ± 3	53 ± 8	0.56 ± 0.39	38 ± 0.1
HF	0.87 ± 0.03	325 ± 92	37 ± 4	132 ± 53	0.59 ± 0.08	104 ± 11
D-GF	1.40 ± 0.06	272 ± 74	59 ± 16	496 ± 42	4.12 ± 0.04	54 ± 9
D-HF	1.60 ± 0.11	690 ± 76	70 ± 5	914 ± 61	12.5 ± 0.12	178 ± 24

Supplementary Table 5. The stored and emitted kinetic energies, and generated electrical energies

Sample	Stored kinetic energy at 1,000 turns m^{-1}	Emitted kinetic energy			Generated electrical energy	
	MJ m^{-3}	MJ m^{-3}	W kg^{-1}	J kg^{-1}	MJ m^{-3}	W kg^{-1}
HF	2.95	0.01	23.38	16.07	0.004	0.05
D-GF	1.44	0.09	145.58	66.72	0.04	0.25
D-HF	2.60	0.20	382.11	125.91	0.08	0.44

Supplementary Table 6. The mechanical properties and densities of various materials

	Tensile strength (MPa)	Shear strength (MPa)	Density (g cm^{-3})	Shear strength / Density	References
GF	229.00	53.00	1.06	50.0	This work
HF	325.00	131.76	0.871	151.3	This work
D-GF	347.53	495.79	1.395	355.4	This work
D-HF	590.22	914.11	1.596	572.8	This work
Aluminum Soft AA3103 H6	175.00	100.00	2.7	37.0	Aalco Metals Limited
Aluminum Soft AA3103 H9	240.00	125.00	2.68	46.6	Aalco Metals Limited
Aluminum Soft AA5251 0	300.00	175.00	2.68	65.3	Aalco Metals Limited
Aluminum Soft AA5083	380.00	210.00	2.7	77.8	Aalco Metals Limited
Aluminum Soft AA2011	395.00	235.00	2.68	87.7	Aalco Metals Limited
Aluminum Soft AA7075 T7	505.00	305.00	2.68	113.8	Aalco Metals Limited
Copper Rolled	220.63	193.05	8.9	21.7	ASM Specialty Handbook
Monel Metal Rolled	517.00	448.16	8.69	51.6	ASM Specialty Handbook
AISI 1018 Mild/Low Carbon Steel 1/4 hard	413.68	310.26	7.85	39.5	ASM Specialty Handbook
Low carbon C.R. Sheet 1/2 hard	496.42	344.74	7.8	44.2	ASM Specialty Handbook
Low carbon C.R. Sheet hard	634.32	420.58	7.75	54.3	ASM Specialty Handbook
40-50% carbon steel	689.47	551.58	7.7	71.6	ASM Specialty Handbook
Steel 1.00 Carbon	-	586.05	7.65	76.6	ASM Specialty Handbook
Steel 1.20 Carbon	-	648.11	7.5	86.4	ASM Specialty Handbook
Silicon	180.00	448.16	2.33	192.3	ASM Specialty Handbook
Stainless (18-8)	860.00	482.63	7.48	64.5	ASM Specialty Handbook
Nickel (3-1/4%)	760.00	565.37	8.9	63.5	ASM Specialty Handbook
Nickel (5%)	795.00	586.05	8.09	72.4	ASM Specialty Handbook
110 Electrolitic Copper -.050 mm GS	220.63	151.68	8.89	17.1	ASM Specialty Handbook
110 Electrolitic Copper – 1/2 Hard	289.58	179.26	8.89	20.2	ASM Specialty Handbook
110 Electrolitic Copper –	344.74	193.05	8.89	21.7	ASM Specialty Handbook

Hard					
220 Comm Bronze 90% – 1/2 Hard	255.00	242.01	8.8	27.5	ASM Specialty Handbook
230 Red Brass 85% – 1/4 Hard	330.95	241.32	8.8	27.4	ASM Specialty Handbook
260 Cartridge Brass – .035 nn Gs	350.00	234.42	8.8	26.6	ASM Specialty Handbook
260 Cartridge Brass– 1/2 Hard	400.00	275.79	8.8	31.3	ASM Specialty Handbook
260 Cartridge Brass – Spring	630.00	330.95	8.8	37.6	ASM Specialty Handbook
Copper 342 A High Lead – 1/2 Hard	413.69	275.79	8.86	31.1	ASM Specialty Handbook
675 Manganese Bronze	448.00	289.58	8.36	34.6	ASM Specialty Handbook
Titanium – Unalloyed	485.00	344.74	4.42	78.0	ASM Specialty Handbook
LDPE	40.00	8.00	0.917	8.7	[S1]
HDPE	45.00	7.00	0.97	7.2	[S1]
PTFE	41.40	3.00	2.2	1.4	[S1]
PP	35.70	12.50	0.946	13.2	[S1]
PMMA	70.00	110.00	1.18	93.2	[S1]
PVC	55.90	48.00	1.3	36.9	[S1]
PS	44.00	65.00	1.04	62.5	[S1]
Jute/Epoxy	36.20	29.21	1.4	20.9	[S2]
Hemp/Epoxy	38.47	37.58	1.6	23.5	[S3]
Nylon6/6	80.00	68.95	1.14	60.5	Poly-Tech Industrial, Inc.
Acetal Copolymer (Delrin®)	68.00	55.16	1.41	39.1	Poly-Tech Industrial, Inc.
Acetal Homopolymer (Delrin®	75.80	62.05	1.41	44.0	Poly-Tech Industrial, Inc.
Delrin® AF Blend	55.16	52.40	1.5	34.9	Poly-Tech Industrial, Inc.
Ertalyte® TX	76.00	58.61	1.44	40.7	Poly-Tech Industrial, Inc.
Fluorosint® 207	10.00	11.72	2.3	5.1	Poly-Tech Industrial, Inc.
Nylatron® GS Nylon	86.18	72.39	1.15	62.9	Poly-Tech Industrial, Inc.
PEEK (Polyetheretherketone)	95.20	55.16	1.23	44.8	Poly-Tech Industrial, Inc.
PET (Thermoplastic Polyester)	85.49	55.16	1.38	40.0	Poly-Tech Industrial, Inc.
Poly-Texx® HPV	89.63	68.95	1.33	51.8	Poly-Tech Industrial, Inc.
Poly-Texx® HPVG	-	75.84	1.33	57.0	Poly-Tech Industrial, Inc.
Poly-Texx® HPVO	-	75.84	1.33	57.0	Poly-Tech Industrial, Inc.
Poly-Texx® HPVT	-	75.84	1.33	57.0	Poly-Tech Industrial, Inc.
Poly-Texx® MDS	72.39	72.39	1.16	62.4	Poly-Tech Industrial, Inc.
Poly-Texx® PVX	129.62	68.95	1.48	46.6	Poly-Tech Industrial, Inc.
Polycarbonate PC 1000	72.39	63.43	1.22	52.0	Poly-Tech Industrial, Inc.
PSU 1000 (Polysulfone)	70.33	62.05	1.23	50.4	Poly-Tech Industrial, Inc.
Radel® R (Polyphenylsulfone)	69.60	62.05	1.35	46.0	Poly-Tech Industrial, Inc.
Semitron® ESd 225	38.00	41.37	1.33	31.1	Poly-Tech Industrial, Inc.
Techtron® PPS (Polyphenylene Sulfide)	93.08	62.05	1.35	46.0	Poly-Tech Industrial, Inc.
Torlon® 4203 (Polyamide-imide)	124.11	110.32	1.41	78.2	Poly-Tech Industrial, Inc.
Torlon 4301 (Polyamide-imide)	82.74	113.07	1.45	78.0	Poly-Tech Industrial, Inc.
Ultem® 1000 (Polyetherimide)	113.76	137.89	1.27	108.6	Poly-Tech Industrial, Inc.
Composite with Carbon Fiber T300	3530.00	201.00	1.76	114.2	[S4]
Composite with Carbon Fiber SYT2	-	269.00	1.8	149.4	Toray Industries, Inc.
Composite with Carbon Fiber T700SC	4900.11	238.00	1.8	132.2	Toray Industries, Inc.
Composite with Carbon T400	2250.00	98.00	1.8	54.4	Toray Industries, Inc.
Composite with Carbon T700	4900.00	136.00	1.8	75.6	Toray Industries, Inc.
Composite with Carbon T800	5880.00	164.00	1.81	90.6	Toray Industries, Inc.
Composite with Carbon T1100	7000.00	160.00	1.79	89.4	Toray Industries, Inc.
Composite with Carbon M60J	3820.00	55.00	1.93	28.5	Toray Industries, Inc.
PAN-based high-strength type 1	3017.09	967.89	1.8	537.7	[48]
PAN-based high-strength type 2	2317.52	995.84	1.8	553.2	[48]
PAN-based high-strength	4146.97	1053.45	1.8	585.3	[48]

type 3					
PAN-based high-strength type 4	2469.18	1104.11	1.8	613.4	[48]
PAN-based high-modulus type 1	2185.09	743.73	1.8	413.2	[48]
PAN-based high-modulus type 2	2363.84	755.07	1.8	419.5	[48]
PAN-based high-modulus type 3	2600.17	795.64	1.8	442.0	[48]
PAN-based high-modulus type 4	2840.68	1109.16	1.8	616.2	[48]
Mesophase pitch-based carbon fiber 1	2033.25	623.72	1.8	346.5	[48]
Mesophase pitch-based carbon fiber 2	2403.10	986.85	1.8	548.3	[48]
Mesophase pitch-based carbon fiber 3	1888.60	973.31	1.8	540.7	[48]

*The density of carbon fibers is assumed at 1.8 g cm^{-3} for comparison.

Supplemental Movie.

Observation of D-HF fiber twisting using optical microscopy.

References

S1. J. K. A. Amuzu, B. J. Briscoe, D. Tabor, Friction and Shear Strength of Polymers. *ASLE Transitions* **20**, 254-358 (1977).

S2. P. Sature, A. Mache, Mechanical Characterization and Water Absorption Studies on Jute/Hemp Reinforced Hybrid Composites. *Am. J. Mater. Sci.* **5,** 133-139 (2015)

S3. M. R. Sanjay, G. R. Arpitha, B. Yogesha, Studies on mechanical strengths of hemp-glass fibre reinforced epoxy composites. *Mat. Today: Proceedings* **2**, 2959-2967 (2015).

S4. H. Fan, Y. Gu, S. Wang, M. Li, Z. Zhang, Characterization and Analysis of Torsion Property of Carbon Fiber Bundle Combined with Epoxy Resin. *Polym. Composite* **39,** E2529-E2539 (2018).
