# Supplementary material for: Carbon nanotube-reduced graphene oxide fiber with high torsional strength from rheological hierarchy control
Source: Nat Commun. 2021 Jan 15;12:396. doi: 10.1038/s41467-020-20518-0 (PMC7810860; doi:10.1038/s41467-020-20518-0)
Supplement: Supplementary file 3 — Description of Additional Supplementary Files [file 41467_2020_20518_MOESM3_ESM.pdf]

Description of Additional Supplementary Files:

Title: Supplementary Movie 1.

Description: Observation of D-HF fiber twisting using optical microscopy.
